# Nosocomial Infections and Role of Nanotechnology

**DOI:** 10.3390/bioengineering9020051

**Published:** 2022-01-28

**Authors:** Thripthi Ananda, Ankita Modi, Ishita Chakraborty, Vishwanath Managuli, Chiranjay Mukhopadhyay, Nirmal Mazumder

**Affiliations:** 1 Department of Microbiology, Kasturba Medical College, Manipal Academy of Higher Education, Manipal 576104, Karnataka, India; anandathripthi@gmail.com; 2 Department of Applied Mechanics, Indian Institute of Technology Madras, Chennai 600036, Tamil Nadu, India; 98ankitamodi@gmail.com; 3 Department of Biophysics, Manipal School of Life Sciences, Manipal Academy of Higher Education, Manipal 576104, Karnataka, India; ishitach1995@gmail.com (I.C.); nirmal.mazumder@manipal.edu (N.M.); 4 Department of Mechanical and Manufacturing Engineering, Manipal Institute of Technology, Manipal Academy of Higher Education, Manipal 576104, Karnataka, India; 5 Center for Emerging and Tropical Diseases, Manipal Academy of Higher Education, Manipal 576104, Karnataka, India

**Keywords:** hospital-acquired infection, nosocomial infection, nanotechnology, healthcare, multidrug resistance

## Abstract

Nosocomial infections, termed hospital-acquired infections (HAIs), are acquired from a healthcare or hospital setting. HAI is mainly caused by bacteria, such as *Acinetobacter baumannii*, *Klebsiella pneumoniae*, *Escherichia coli*, *Enterococci* spp., Methicillin-resistant *Staphylococcus aureus* (MRSA), and many more. Due to growing antibacterial resistance, nanotechnology has paved the way for more potent and sensitive methods of detecting and treating bacterial infections. Nanoparticles have been used with molecular beacons for identifying bactericidal activities, targeting drug delivery, and anti-fouling coatings, etc. This review addresses the looming threat of nosocomial infections, with a focus on the Indian scenario, and major initiatives taken by medical bodies and hospitals in spreading awareness and training. Further, this review focuses on the potential role nanotechnology can play in combating the spread of these infections.

## 1. Introduction

The term ‘nosocomial’ is derived from two Greek words, ‘nosus’ and ‘komeion’ that literally translate into ‘disease’, and ‘take care of’. It was during the first half of the 18th century that the scientific study of nosocomial infection or hospital-acquired infection (HAI) started [1]. During the post-World War II era, various antibiotics were discovered and widely used in treatment. Penicillin was used extensively, which caused the death rate due to postoperative pneumonia to reduce from 30% to <10% and surgical wound infections to reduce below 5%. By the 1960s, the surgical infection rate reduced below 2% due to the introduction of other antibiotics [2]. However, this soon led to a major penicillin-resistant Staphylococcal epidemic amongst patients and health care workers and many of them were nasal and dermal carriers [3]. After the discovery of methicillin and vancomycin in the 1960s, the emergence of methicillin-resistant *Staphylococcus aureus* (MRSA) began due to overuse. Along with MRSA, other resistant organisms that emerged were vancomycin-resistant *Enterococcus* spp., a few members of the Enterobacteriaceae family, *Pseudomonas aeruginosa*, *Streptococcus* spp., and *Candida* spp. Hence, nosocomial infections are infections that occur in a patient while receiving care in a hospital or other health care facility [4]. After an infection is confirmed to be of nosocomial origin, according to the definition mentioned before, the specific type of infection is categorized based on the systemic classification provided by the National Health Safety Network (NHSN) with the Centers for Disease Control and Prevention (CDC) which are specifically based on clinical and biological criteria [5]. According to the NHSN and CDC criteria, HAIs have been classified into 14 different types. Out of these, the incidences of device associated HAIs (DA-HAI) are the most common in healthcare settings which include central-line associated bloodstream infections (CLABSI), catheter-related bloodstream infections (CRBSI), catheter-associated urinary tract infections (CAUTI), ventilator-associated pneumonia (VAP), and surgical site infections (SSI) [6]. The most common nosocomial infection-causing bacteria include *S. aureus,* including antibiotic-resistant MRSA, *Escherichia coli*, *Enterococcus* spp., and *Candida* spp. With the increase in antibiotic resistance of nosocomial infections, nanoparticles, especially metallic nanoparticles, provide a successful alternative due to their special properties including high reactivity and stability. Nanoparticles increase the permeability of bacterial cell membranes, resulting in a higher uptake of antibiotics by the bacterial cells [7].

It is estimated that out of every 100 inpatients in a health care setting, 10 and 7 patients can get infected by HAI in developing and developed countries, respectively [8]. Various multicentric studies around the globe revealed the prevalence of hospitalized patients to acquire at least one HAI ranged from 3.5 to 12% [9,10]. The most common causes of nosocomial infections include the use of catheters, surgical procedures, and mechanical ventilation.

**Figure 1:**
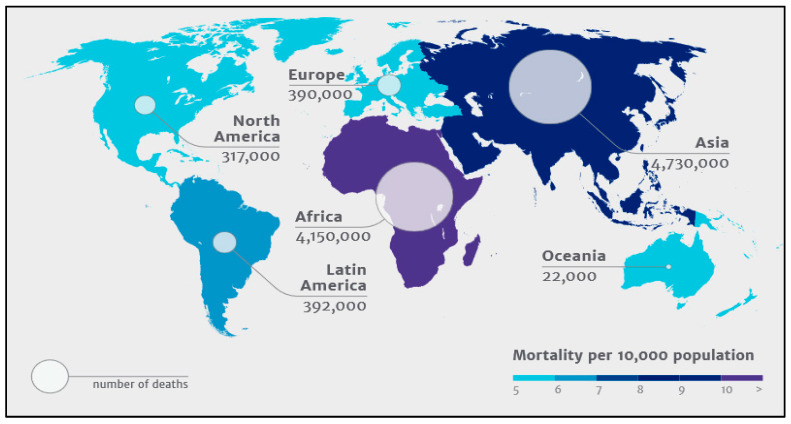
Global distribution of 10 million deaths expected by 2050 due to antimicrobial resistance. (Source: The Review on Antimicrobial Resistance–Tackling drug resistant infections globally, The Wellcome Trust, The UK Department of Health, London, 2014).

Globally, the incidence of HAI ranges from 3.6 to 19.1%. Out of these, high-income countries (HICs) account for 3.6–12%, whereas the low- and middle-income countries (LMICs) account for 5.7 to 19.1% [11]. Studies state that the prevalence rate is up to 4.5% in the US and 5.7–7.1% in European countries, while this rate ranges between 5.7% and 19.2% in low- and middle-income countries [12,13,14,15]. Based on an extensive study conducted by the WHO in 2011, HAI incidence density ranged from 13.0 to 20.3 episodes per 1000 patient-days in the US and Europe, respectively [11]. While more than 40% of hospitalizations with HAI were observed in Latin America, Sub-Saharan Africa, and Asia [16]. The pooled cumulative incidence density of HAI (HAI/1000 patient-days (95%CI)) in HICs and LMICs are 17.0% (14.2–19.8%) and 42.7% (34.8–50.5%), respectively. As per an independent review by a UK committee, it is assessed that 10 million deaths will happen by 2050. Figure 1 shows the global distribution of the same.

The various risk factors influencing HAI depend on the environment where the care is given, susceptibility of the etiological agents, patient condition, and lack of knowledge on the existence and prevalence of such infections amongst staff and health care providers in a health care setting. Infectious agents keep shedding from infected or carrier patients or health care workers and settle at various sites within the health care settings, where these agents remain viable for days risking infections to other patients and staff [17,18,19]. With increasing trends of resistance in these pathogens, there are limited antibiotics that can be used to treat these infections. In a few instances, the bacteria become resistant to all the available antibiotics, increasing the challenges to patient treatment and adding to the cost of management. Various technologies and other innovative strategies have been adopted and are under trial to combat and prevent infections from resistant organisms. Nanotechnology has gained interest amongst researchers due to its ability to improve existing therapeutics, by enhancing the physiochemical properties and stability of antibiotics, inhibiting or minimizing biofilm growth, target delivery at the infection site, and reducing side effects [20]. It includes the use of nanoparticles of various elements to deal with the current HAI issues. For example, to combat nosocomial infections as drug delivery systems, molecular beacons, and to control biofilm formations. This review summarizes the looming threat of nosocomial infection in the Indian subcontinent and common applications and recent advances of nanotechnology against nosocomial infections.

## 2. Indian Scenario

A narrative review from India reported the incidence of HAI within the range of 4.4 to 83.09% [21]. A compilation of various studies indicated that the overall incidence of SSI and CRBSI in India ranged from 2 to 21% and 0.2 to 28%, respectively, with a rate of 0.5–47 per 1000 catheter days [21,22]. *E. coli* and *Staphylococcus* spp. were the common etiological agents isolated from DA-HAI. In 2014, International Nosocomial Infection Control Consortium (INICC) presented the brief SSI rates of 7 years from six major cities in India, according to which, the highest SSI rates were observed in breast surgery (8.3%) followed by exploratory abdominal surgery (6.0%) [23]. A six-year multicentric study data on peripheral venous catheter-related bloodstream infection (PVCR-BSI) rates from 24 Intensive care units (ICUs) in 19 cities in India was published by INICC in 2020 [24]. A total of 863 Peripheral Venous Catheter-Related Bloodstream Infections (PVCR-BSI) were identified with a rate of 2.91/1000 PVC days. The mortality rate in patients with PVCR-BSI was 11.59%, while that in patients with PVC but without PVCR-BSI was 4.14%. The length of stay was greater in patients with PVCR-BSI (6 days) when compared to that of patients with PVC but without PVCR-BSI (4 days). Gram-negative organisms (68.0%) were the major causative agent, which includes *E. coli* (23.0%) and *K. pneumoniae* (15.0%), while *S. aureus* (10.0%) was the predominant Gram-positive organism.

Various studies have been conducted in India tracking the HAI rates, etiological agents, and its risk factors. In 2018, according to a multicentric study, which included the southern, western, and northern states of India, showed that VAP (58.5%) was the most common HAI amongst ICU patients, followed by CRBSI (21.2%) with the highest mortality observed in CRBSI (34.6%), followed by VAP (26.0%) [25]. *K. pneumoniae* was the most commonly isolated etiological agent, but the highest resistance was observed in *Acinetobacter baumannii* (87.5%). More than 50% resistance was observed in *K. pneumoniae*, *E. coli*, and *P. aeruginosa*. Studies conducted in southern India also showed that VAP is the most common HAI amongst hospitalized patients ranging from 23–51% of the total HAI infections [26,27,28]. *K. pneumoniae* (19.0–24.0%), *A. baumannii*, and *P. aeruginosa* (both 11.0–25.0%) were commonly isolated in VAP, but *A. baumannii* and *P. aeruginosa* were statistically significant for the same. While *A. baumannii*, *P. aeruginosa*, and *S. aureus* were accounted for in CRBSI, organisms belonging to the Enterobacteriaceae family, especially *E. coli* (26%), commonly cause CAUTI. It was observed that *P. aeruginosa* showed an increasing trend in resistance for piperacillin-tazobactam, amikacin, and imipenem, while other non-fermenters showed increased resistance to most antibiotics, except for imipenem (33.1%) and polymixin B (2.4%) [28]. Increased hospital stays, a high acute physiology and chronic health evaluation (APACHE) III score, and chronic kidney disease was significantly associated with mortality. Studies conducted in the neonatal intensive care unit (NICU) showed that BSI was the most common HAI amongst neonates which is up to 80%. The etiological agents were similar to that of adults with extended-spectrum beta-lactamase (ESBL) *K. pneumoniae* and MRSA being the common resistant pathogens [29]. Low birth weight, premature birth, and increased hospital stay are the risk factors amongst neonates. These results were similar to the studies conducted in NICUs in northern India with BSI (73%) as the major HAI, followed by pneumonia (12%), with low birth weight and premature birth as the risk factors, and peripheral vascular catheters and ventilators as the device-related risk factors [30]. MRSA exhibits high resistance to penicillin, ciprofloxacin, and erythromycin, while *A. baumannii* and members of the Enterobacteriaceae family showed resistance to most of the traditional high-end antibiotics. Other studies in northern India revealed that the incidence rate of HAI lay between 18.3–28.6/1000 patient days with VAP being the common DA-HAI amongst adult hospitalized patients [31,32,33]. The incidence of VAP, CRBSI, and CAUTI rates in hospitalized patients was around 11–22, 2–8, and 8–10 per 1000 device days. The duration of use of medical devices was higher amongst patients with DA-HAI compared to the patients without HAI. *A. baumannii* (24–40%) and *K. pneumoniae* (15–28.5%) were the most common etiological agents causing DA-HAI with *A. baumannii* being predominant in VAP [33,34]. Additionally, these causative agents showed high resistance to carbapenems (85–100%). Studies conducted in the western part of India also showed that VAP (17.44- 27.0%) was the common HAI amongst hospitalized patients, followed by CRBSI and CAUTI with an incidence rate of 21.9, 0.48, and 0.6 per 1000 patient days [35,36,37]. The use of mechanical ventilators, urinary catheters, and prolonged hospital stays were the common risk factors leading to acquiring HAI. *S. aureus* and *K. pneumoniae* were the common etiological agents leading to HAI, with *K. pneumoniae* leading in VAP [35]. The increased incidence of HAIs is affecting patient care and questioning the various implemented measures, technology, and strategies adopted to deal with the causative agents.

Infection control guidelines and strategies started very early in India, but they lacked proper training and knowledge amongst the healthcare workers [38]. In 2012, the Indian Council of Medical Research (ICMR) had introduced Antibiotic Stewardship, Prevention of Infection & Control (ASPIC) which was designed to get together faculties from clinical pharmacology, microbiology, and other disciplines to associate together, plan, and improve antibiotic stewardship and control and decrease hospital infections through constructive infection control practices [39]. Twenty centers throughout the country participate in this program per year and come together for a training workshop. In 2020, an ASPIC meeting was held where ideas on nanoscience and phage therapy were discussed as methods to deal with resistant organisms and nosocomial infections [40]. In 2017, a study was carried out in south India where the impact of the “care bundle approach” was analyzed, which helped reduce the incidences of DA-HAIs [41]. The care bundle comprises the necessary elements of care procedures for enhanced and systematic monitoring of the treatment and care of patients. The “care bundle” approach has effectively helped to manage DA-HAIs [41,42]. A major step to control these infections is the education, training, and creation of awareness amongst health care workers, especially nurses [43,44]. The healthcare workers can help reveal prevailing practices and issues as well as propose measures concerning hospital hygiene and HAI prevention and control practices; these suggestions can help in building future structural and behavioral interventions against HAI [45]. The positive impact has been observed amongst healthcare workers in studies based on educational and training programs on HAI, standard precautions, and hand hygiene [46,47]. It is also observed that the systemic surveillance of HAI in India can help in strengthening infection prevention and control [48].

## 3. Nano Strategies Combating Nosocomial Infections

The past decade has seen a continuous focus on developing new strategies and therapeutic innovations to handle Multi-drug Resistant (MDR) strains in the post-antibiotic era. Nanotechnology has emerged as a potential savior. According to the BCC survey, the global market for healthcare-acquired infection (HCAI) will increase from $18.9 billion in 2018 to $24.7 billion by 2023, while the global nanomedical market will grow from a whopping value of $151.9 billion in 2017 to $293.1 billion by 2022 [49,50]. From its conceptual introduction in the 1960s by Prof. Richard Feynman to its envisioning of applications in molecular biology and medicine by Drexler [51], the influence of nanotechnology has grown exponentially in many sectors. This scientific literature survey indicates that various fields of medicine would be benefited from nanotechnology in the near future and that it will spread rapidly in many other domains.

The development of antibiotic resistance is a rising crisis today. Further, genetic tolerance against antibiotics in bacteria such as MRSA is a common occurrence. Nanomaterials inhibit bacterial growth or activity that results in infections. Nanoparticles penetrate the bacteria and biofilm leading to reactive oxygen species (ROS) generation that eliminates bacteria. Thus, nanoparticles are a novel approach to combat drug-resistant bacterial infections. Different nanomaterials, such as nanoparticles and nanotubes, are directly used in biomedical devices to prevent spreading infections.

**Figure 2:**
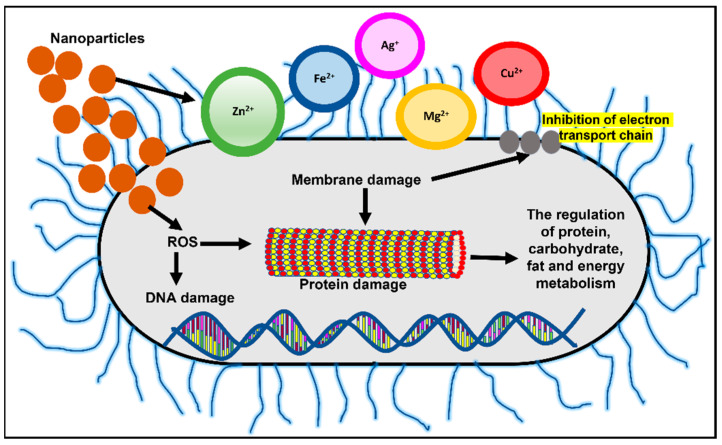
Mechanisms of nanoparticle action in bacterial cells include changing membrane permeability and the generation of free radicals leading to DNA and protein damage. Adapted from Wang et al., 2017, Copyright 2017 Informa PLC [56].

Owing to the small size and high surface area to volume ratio, nanoparticles (NPs) have a much stronger physical interaction capability with bacterial cells compared to microparticles. These NPs offer many interaction mechanisms between nanomaterials and cell walls, such as changing the membrane permeability by penetration, blocking oxidative phosphorylation, or by generating free radicals leading to damage of the cell membrane, and in turn cell death, thus increasing the oxidative stress and destroying DNA [52], as shown in Figure 2. Additionally, the ionic activity of nanoparticles can modulate the bacterial signal transduction leading to the inhibition of bacterial growth or inactivating the enzymes by interacting with them. There are physical interaction mechanisms too, which include bacterial wrapping to induce surface stresses and penetration through sharp edges that causes physical damage and adverse chemical effects [53,54]. Similarly, helpful is the creation of anti-adhesion surfaces that inhibit biofilm formation [55]. In the present review, an attempt was made to highlight the potential novel solutions provided by nanotechnology in the diagnosis, treatment, and control of MDR strains and its potential use in combat against nosocomial infections.

### 3.1. Nanoparticles Based Molecular Beacons

**Figure 3:**
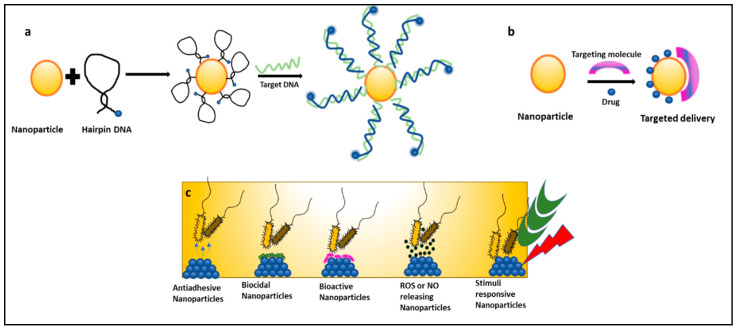
Mechanisms of nanoparticles combating nosocomial infections: (**a**) nanoparticles as molecular beacons, (**b**) nanoparticles for targeted drug delivery of antibiotics, and (**c**) types of nanoparticles preventing biofilm-associated nosocomial infections.

Nanoparticles can be used as molecular beacons to identify relevant bacterial strains in minimal time [57,58,59,60,61,62]. This is especially important in emergency cases or in an ICU where immediate results are required to continue medical procedures. Molecular beacons are the most promising method for qualitative and quantitative biological detection of bacteria. In a recent study, molecular beacons loaded with nanoparticles have been used for the rapid detection of bacteria and viruses (Figure 3a). In another recent study, it was observed that hybridizing molecular beacons with nanoparticles improved the bacterial efficiency. The new age nanoprobes comprised of molecular beacons hybridized to gold nanoparticles could detect *E. coli* at a concentration of 10^2^ cfu/mL. This method was 1000 times more sensitive than detecting using only molecular beacons [57].

Various NPs are employed in life science to accurately detect a bacterial strain using the following techniques: Surface-enhanced Raman Spectroscopy (SERS) detection method, the rapid and cost-effective immuno-magnetic separation technique, and short detection time-based fluorescence microscopy, as well as fast and visual methods like the calorimetric method or highly sensitive methods like real-time PCR techniques [52,58,59]. In sandwich-structure immunoassays, *E. coli* and *S. aureus* were detected using the SERS method in a total assay time of 10 min [60]. Similarly, in another study, *E. coli* and *S. epidermidis* were detected using the SERS method in 10 min by employing the synthesis of AgNPs coating on the cell wall of bacteria [61]. This leads to the 30 times increase in the Raman signal of these bacteria compared to that obtained using a mixing of colloid and bacterial suspension. Additionally, reports are showing the presence of cost-friendly techniques to detect bloodstream infection using magneto fluorescent NPs which proved to be time-saving and equally sensitive methods [62]. Apart from this, the inherent superiority of molecular beacon probes and biofunctionalized NPs led to a series of novel principles, methods, and techniques to exploit bioanalytical and biomedical studies as well.

### 3.2. Nanoparticles Formulated with Drugs and Antibiotics-Nano Bactericidal

Nanomedicine is an offshoot of nanotechnology under exploration for its suitability in the delivery of treatment and healthcare benefits. It provides platforms like targeted drug delivery using different nanoparticulate systems, a multidrug complex entrapped in a single nanoparticle, surface functionalization with nano-particulate antibiotic materials, nano antibiotics, and creating ROS using inorganic metal oxide NPs, playing important roles in the restoration of antibiotic activity (Figure 3c) [63,64]. Antibiotics conjugated with NPs give the advantage of lower sample consumption and higher sensitivity [65]. Studies have reported that nanoparticles coupled with antibiotics, such as vancomycin, amoxicillin, and penicillin G, show an enhanced antimicrobial resistance against *S. aureus* and *E. coli* [66]. NO (Nitric oxide) releasing nanoparticles and metal oxide nanoparticles (TiO_2_, ZnO) have also shown effective antimicrobial activity against many MDR strains and are under exploration [52]. In a recent study, gold nanoparticles (Au NPs) were conjugated with ceragenin CSA-131 (cationic steroid antimicrobial) and were tested for their bactericidal activity against *S. aureus*, *S. epidermidis*, *K. pneumoniae*, *K. oxytoca*, and *P. aeruginosa*. All nanosystems exhibited potent bactericidal activity by generating ROS, resulting in the damage of the bacterial membranes and the leakage of intracellular content [67]. Researchers have taken an interest in exploring the suitability of nano-photo thermal therapy for MDR bacteria. This therapy involves the selective killing of bacteria by the transfer of heat generated from the conversion of electromagnetic radiation. It irreversibly damages the bacterial membranes and interferes with cell wall biosynthesis. These outcomes are a boon to researchers as antibiotics are turning out to be inefficient [67,68].

### 3.3. Nanotechnology in the Development of Drug Delivery Systems (Nano-DDS)

Drugs with poor solubility and absorption ability can be delivered via nanoparticles for target-specific drug delivery. However, the efficacy of these nanoparticles for drug delivery depends on factors such as size, shape, and other physical/chemical characteristics [20]. Malachite green (MG) encapsulated in mesoporous silica nanoparticles (MSN) was tested against common nosocomial infection-causing bacteria such as *S. aureus* and *E. coli*. MG-MSN was found to be effective against both tested bacterial strains. *S. aureus* was more phototoxic to MG-MSN compared to *E. coli*. The anti-biofilm efficacy of MG-MSN on *E. coli* and *S. aureus* were also studied. Biofilm inhibition was found to be 65.68 ± 2.62% in *E. coli* and 79.66 ± 3.82% in *S. aureus* [69]. Nano-DDS is a promising approach in the control of HCAI, but only a small number of nano-DDS products were commercially successful in the market [70,71]. This is mostly due to financial profitability and poor information about product functioning available to health professionals across countries, leading to further delay in its full exploitation concerning generic medicine. Similarly, concerns about the toxicity of nanoparticles, their effect on the blood-brain barrier, and their delivery to the central nervous system are limiting their application in medicine. Industrial production, quality control, and storage stability of nanoparticles must be assessed to ensure their purity and safe administration in a biological system [72]. Nevertheless, the progress of the research in this domain promises a higher success ratio in defending the patient’s interests.

### 3.4. Surface Modifications to Control Biofilm-Associated Infections

Biofilm-based infections are important causes of morbidity, affecting millions of people every year, typically causing chronic nosocomial infections. The current clinical practice with biofilm-associated infection is to treat it with high-dose antibiotics and if symptoms persist, a surgical replacement can be done to reduce further complications to patient health [73]. Tailoring the functional surface properties of implants or biomedical devices used during treatments can curb the initial and later stages of infection development. Nanoparticles facilitate the sustained release of attached bioactive materials or ions and thus provide longer antibiofilm activity. In a study conducted on the antibacterial activity of silver nanoparticles (AgNPs) against nosocomial *A. baumannii* AIIMS 7 in biofilm mode, nanoparticles exhibited significant biofilm disruption activity at a minimum inhibitory concentration of 2 mg/mL. The eradication of the biofilm was improved on combined exposure to AgNPs and antibiotics. These nanoparticles inhibited bacterial growth through intracellular oxidative stress and interact with thiol-groups in cellular proteins resulting in denaturation [74]. Similarly, surface modification of other nanoparticles such as AuNPs makes them desirable to be used for oral biology [75] and other healthcare applications. A detailed study on various types of nanoparticles and their results are tabulated in Table 1.

Methodologies like anti-fouling, anti-adhesive or bactericidal coating, and many more methods are adopted for reducing bacterial adhesion on medical devices [73,76]. These multifunctional coatings simultaneously promote osseointegration and prevent infection of implants. Silver is the most common bactericidal agent used to date due to its broad spectrum of antimicrobial activity against both Gram-positive and negative bacterial strains [76]. The inorganic nanoparticles of Ag and Au were used in coating urethral [77], venous and ventricular catheters [78], organic nanoparticles based on chitosan and PEG stabilized lipid in bone and dental implants [79], as well as several other metallic/metal-polymer composites in the development of face masks, heart valves, pedicle screws, contact lenses, and orthopedic and oral implants depending on a working mechanism [80]. Table 1 exhibits the studies regarding the antibacterial applications of nanostructures against common nosocomial infection causing bacterial strains.

**Table 1:** Antibacterial applications of nanostructures.

Study	Outcome of Study	Reference
In-vivo study of different nanostructured surfaces		
Effect of nanoporous features on titanium screw implants in rat femurs. Features were created using anodizing process.	No sign of infection in 28 days’ test over nano-porous surfaces. A sign of infection was found around un-anodized nano-smooth titanium implants.	[81]
Effect of nano-roughness on silicon nitride material implant in rat skull.	Studies were conducted for 28 days’ test with or without bacteria. No sign of infection was observed on the nano-roughened surface while significant *P. aeruginosa* was observed on the smooth silicon nitride material implant.	[81]
Analyzing antimicrobial and antibiofilm properties of ZnO nanorods decorated with graphene nanoplatelets against dental pathogens.	Cell viability assay and Filed Emission-SEM analysis showed the attainment of high killing rates of *S. mutans* cells and visible physical damages over the cell surfaces due to nanorods. Safranin assay showed a 30% reduction in biofilm development.	[82]
Studies on various surface modification		
Silver plasma immersion ion implantation (Ag-PIII) over the implant surface leads to the embedment of AgNPs over the surface.	Bactericidal efficacy against relevant bacterial species was shown as well as promoted osteogenesis both in vitro and in vivo. A 99% reduction in viability for *S. aureus* was observed.	[83]
Coating of modified Nano TiO_2_ on a solid surface to create an antimicrobial film over it.	Light fall on a coated surface generates the electron-hole pairs which promoted the death of microbial cells. The study showed inhibition of *E. coli*, *S. aureus*, and *P. aeruginosa* strains. This result proposes a promising, long-lasting, and effective technique against the nosocomial environment.	[84]
Titanium substrate surface engineered with Chitosan for functional Ti-based orthopedic implants.	A chitosan-lauric acid (Chi-LA) conjugate showed a 95% and 93% antibacterial efficacy against *S. aureus* and *P. aeruginosa* over 1 week as well as the modified surface-enhanced biological functions of osteoblasts and concurrently reduced bacterial adhesion.	[85]
AgNP/poly(DL-lactic-co-glycolic acid) (PLGA)-coated stainless steel alloy (SNPSA) as a potential antimicrobial implant material.	In vivo experiments showed that after 8-weeks no bacteria (*S. aureus* and *P. aeruginosa*) and minimal inflammatory cells were found in tissue surrounding the implant. SNPSA exhibited strong bactericidal and osteoinductive properties.	[86]
Antifouling Coating studies		
Creating an inert polymer brush layer on the surface using polyethylene glycol (PEG).	The surface reduced the level of adhesion of *P. aeruginosa* by 2–4 orders of magnitude up to 5 h.	[87]
Titanium surfaces were modified with poly(methacrylic acid) (P(MAA)).	MAA reduced adhesion of *S. aureus* and *S. epidermidis* by 50%.	[88]
Titanium Nanotubes anodized with silver nitrate to provide antimicrobial efficacy.	The study showed that *P. aeruginosa* viability was decreased one thousand-fold on the nanotubes while supporting osteoblast cell adhesion.	[89]
Created nanotube array over Ti substrate. Measured the antimicrobial and osteogenic properties.	Smaller nanotubes supported better adhesion of osteogenic cells while maintaining the opposite trend in *S. epidemidis* adhesion.	[90]
Created densely packed vertical titania nanocolumns on Ti6A14V surface.	The arrangement of these columns had minimal effect on the attachment of osteoblasts while significantly reducing the biofilm formation of multiple clinical *S. aureus* strains.	[91]
Zinc loaded titania nanotube was used to prevent infection and enhance osseointegration.	Zn-loaded nanotubes increased osseointegration in vivo in rodent tibial insert model and inhibited *S. aureus* growth.	[92]

To address the looming threat of nosocomial infection spread, there is an urgent need for a comprehensive action plan at the national and international levels. An independent review by a committee sponsored and supported by Wellcome Trust and the Department of Health, UK provides a deeper insight and solutions. HAI account for 0.7 million deaths globally, causes the death of nearly 60,000 newborns in India each year, two million infections in the USA alone, and caused 20 billion USD in excess costs. Additionally, the death toll may increase to 10 million by 2050. Therefore, the committee came up with a suggestion of tackling antimicrobial resistance on ten different fronts [93]. The list includes Public Awareness, Surveillance, Human Capital, Vaccines and Alternatives, a Global Innovation Fund, Rapid Diagnostics kits to guide doctors, and an International Coalition for action, etc. In this way, nanotechnology can play a major role and offer solutions in rapid diagnostics, control of the spread, and vaccine effectiveness.

## 4. Conclusions

The increased emergence of antimicrobial resistance has forced us to look for alternative solutions to antibiotics. Nanotechnology has helped in developing multifunctional nanomaterials that offer a number of solutions to counter the nosocomial infection problem in the world and are promising alternatives. Its broader use can help in reducing nosocomial infection cases in the health care sector. Currently, some bottleneck issues are withholding the full-scale utilization of solutions offered by nanotechnology, such as potential toxicity and the effect on the normal functioning of cells on long-term exposure to nanoparticles with a smaller diameter. Issues related to MDR strains develop resistance to these novel technological solutions and consequences of it, but all these issues are addressable. Despite various signs of progress, nanotechnology-based solutions are not widely accepted in diagnosis, treatment, and control because of their expensive nature, poor information about product functioning for health professionals, and concerns among patients. Additionally, there are high chances of deviation in results when the same NPs are used that are successful in treating MDR strains in vitro. All these indicate research failures in analyzing the requirements, effects, and results of in vivo and vitro environments.

The extent of attention and speed at which research is happening in nanomaterials, and the biophysical modification of live tissue exposed surfaces to control the infection spread, is a positive hope. Currently, the toxicity and pharmacokinetics of nanoparticles in in vivo applications are the concern, in addition to the requirement of larger clarity about interactions between coating surfaces, biofilms, and hosts. These need an additional investigation before clinical application. To obtain better and universally acceptable treatment solutions against MDR strains, there is a need for united efforts from nano engineers and microbiologists. Once obtained, it will pave the path for successful implementation in the clinical sector at a larger scale. In addition to this, cost-efficient alternatives need to be identified to benefit humanity. This literature review attempts to create awareness about the larger threat of nosocomial infections and the potential role of nanotechnology in overcoming it.

## Data Availability

No new data were created or analyzed in this study. Data sharing is not applicable to this article.
